# Intraoperative Neuromonitoring of the Visual Pathway in Asleep Neuro-Oncology Surgery

**DOI:** 10.3390/cancers15153943

**Published:** 2023-08-03

**Authors:** Christos Soumpasis, Alba Díaz-Baamonde, Prajwal Ghimire, Asfand Baig Mirza, Marco Borri, Josef Jarosz, Richard Gullan, Keyoumars Ashkan, Ranjeev Bhangoo, Francesco Vergani, Jose Pedro Lavrador, Ana Mirallave Pescador

**Affiliations:** 1 Neurosurgical Department, King’s College Hospital Foundation Trust, London SE5 9RS, UKk.ashkan@nhs.net (K.A.);; 2 Department of Neurophysiology, King’s College Hospital Foundation Trust, London SE5 9RS, UK; 3 Department of Neuroradiology, King’s College Hospital Foundation Trust, London SE5 9RS, UKjozef.jarosz@nhs.net (J.J.)

**Keywords:** optic radiation, visual evoked potentials, intraoperative neuromonitoring, intra-axial tumour

## Abstract

**Simple Summary:**

To optimise patient outcomes in tumour surgery in visual eloquent areas, it is crucial to preserve the integrity of the visual pathways. The aim of our prospective study is to examine the use of visual evoked potentials (VEPs), utilising different techniques such as transcranial and direct cortical recording. In a cohort of 39 patients, we established a significant correlation between the infiltration of the optic radiation by the tumour on tractography and the occurrence of visual field deficits after surgery. In contrast to the transcranial recordings, direct cortical VEP recordings exhibited a robust correlation with visual outcomes. Lastly, a 40% decrease in the amplitude of the N75 and P100 waves of the direct cortical recordings was associated with the risk of a worsening visual outcome. VEP monitoring can be a reliable method to detect visual deficits, but there is a need to improve the technique as it is prone to false warnings.

**Abstract:**

Brain tumour surgery in visual eloquent areas poses significant challenges to neurosurgeons and has reported inconsistent results. This is a single-centre prospective cohort study of patients admitted for asleep surgery of intra-axial lesions in visual eloquent areas. Demographic and clinical information, data from tractography and visual evoked potentials (VEPs) monitoring were recorded and correlated with visual outcomes. Thirty-nine patients were included (20 females, 19 males; mean age 52.51 ± 14.08 years). Diffuse intrinsic glioma was noted in 61.54% of patients. There was even distribution between the temporal, occipital and parietal lobes, while 55.26% were right hemispheric lesions. Postoperatively, 74.4% remained stable in terms of visual function, 23.1% deteriorated and 2.6% improved. The tumour infiltration of the optic radiation on tractography was significantly related to the visual field deficit after surgery (*p* = 0.016). Higher N75 (*p* = 0.036) and P100 (*p* = 0.023) amplitudes at closure on direct cortical VEP recordings were associated with no new postoperative visual deficit. A threshold of 40% deterioration of the N75 (*p* = 0.035) and P100 (*p* = 0.020) amplitudes correlated with a risk of visual field deterioration. To conclude, direct cortical VEP recordings demonstrated a strong correlation with visual outcomes, contrary to transcranial recordings. Invasion of the optic radiation is related to worse visual field outcomes.

## 1. Introduction

Brain tumour surgery poses significant challenges to neurosurgeons due to the intricate relationship between the tumour and adjacent eloquent brain areas. Preserving critical neural structures, such as the visual pathways, is of utmost importance to minimise postoperative complications and optimise patient outcomes [1]. Resection of lesions in the occipital, parietal and temporal lobes is associated with a risk of visual deficits due to their proximity to the visual pathway [2]. DTI data (diffusion tensor imaging) can help identify lesion-induced displacement of the tracts and plan a safer surgery [3].

Visual acuity during surgery can be monitored either by monitoring the visual fields (VF) in an awake setting or by monitoring the visual evoked potentials (VEPs). Studies in awake craniotomies demonstrate that direct monitoring of the VF of the patients can prevent hemianopia [4,5,6,7]. In recent years, the integration of real-time intraoperative monitoring of VEPs in brain tumour surgery has shown promising results [8,9,10].

VEPs are typically recorded using scalp electrodes and represent the summation of synchronised neural activity in the visual pathways. The main component of interest in VEPs is the P100 wave, which corresponds to the activation of the primary visual cortex. Variations in the latency or amplitude of the P100 wave can indicate a functional compromise in the visual system. While transitory VEP changes indicate a reversible visual deficit, permanent VEP changes may translate into a permanent deficit [11].

VEP abnormalities are apparent in most patients with tumours that affect the visual pathway, including the optic radiation and the visual cortex, even when the visual acuity is preserved [12,13]. Multiple attempts have been made in the past to classify visual eloquent gliomas based on their anatomical relationship with optic radiation [3]. However, a functional classification based on intraoperative VEP integrity is lacking. VEPs provide valuable insights into the functional integrity of the visual system during brain tumour resection, enabling surgeons to make informed decisions and enhance surgical onco-functional balance.

Multiple studies indicate that VEP monitoring can often be unreliable. VEPs from transcranial recordings are sensitive to anaesthetic changes [14,15,16,17]. Other factors, such as the craniotomy, the intraoperative brain manipulation during tumour resection, and the invasion of the visual pathway from the tumour, also play a significant role in the reliability and reproducibility of the recordings [18,19].

Ota et al. (2010) demonstrated reliable detection of visual responses via a direct cortical recording in a small population of patients that underwent epilepsy surgery under general anaesthesia [20]. Eleven years later, Boëx et al. showed in a mixed population of neuro-oncology and neuro-vascular patients that subcortico–cortical evoked potentials can be observed in the optic radiation and Meyer’s loop [9]. They correlated the latency of the waveforms to the velocity of signal propagation along the optic pathway. More recently, Carrai et al. (2023) demonstrated in a cohort of 10 patients that direct cortical recordings show better stability of the waveforms in comparison to transcranial recordings [10].

This study aims to explore the application of VEPs in brain tumour surgery, highlighting their significance and clinical outcomes. We utilise different techniques of VEP monitoring, including transcranial and direct cortical recording, when feasible. We aim to distinguish which method is best correlated with functional visual outcomes postoperatively.

## 2. Materials and Methods

This is a single-centre prospective cohort study conducted from January 2021 to December 2022. Patients of age ≥ 18 years old admitted for surgery due to intra-axial post-chiasmatic tumours within the visual eloquent areas (temporal, parietal and occipital lobe) were enrolled.

Preoperative tractography was performed using Stealth Viz Medtronic© software S8 with a diffusion tensor imaging model (DTI). Two regions of interest (ROI) were defined: a start ROI in the lateral geniculate body and an end ROI at the calcarine fissure. An FA value of 0.18 and a maximal angulation of 45 degrees were used in the model.

All of the patients in our cohort underwent general anaesthesia in the form of TIVA (total intravenous anaesthesia), using Propofol and Remifentanil. No use of inhalation agents was made. The depth of anaesthesia was continuously monitored by the neurophysiology team through direct analysis of the raw EEG recording and supported by the CSA (Compressed Spectral Array) index. Both parameters provided an indication of the level of unconsciousness during surgery. Frequent communication between the Anaesthesia and Neurophysiology teams allowed them to adjust the anaesthesia depth, avoiding burst suppression.

Visual function was monitored by stimulating the eyes unilaterally or bilaterally with red or white LED flash goggles or pads. Electroretinography (ERG) was constantly recorded in order to check for the appropriate functionality of the stimulating electrode. Scalp corkscrew electrodes were placed on O1, O2, Oz, Cz-, A1 and A2, according to the International 10–20 EEG system and placement guidance using the intraoperative stealth system. O1-Cz/Fz, O2-Cz/Fz, O1-A1, O2-A2 and O1/2-Oz scalp recordings were taken. Peaks N75 and P100 (ISCEV standards) were monitored intermittently throughout the procedure and compared to the patient’s intraoperative baselines.

Whenever deemed safe, depending on the location of the tumour and the mass effect, a strip electrode (4–6 contacts) was placed along the longitudinal fissure after the dura opening, parallel to the falx cerebri and onto the visual cortex (interhemispheric location, perpendicular to the calcarine fissure). The position was confirmed under direct vision or with intraoperative ultrasound. This strip electrode was used for a continuous direct cortical recording of the N75 and P100 waves. We used a warning threshold of a 50% decrease in amplitude and a 10% increase in latency as an intraoperative alarm for potential neurological damage [18].

The corrective actions were undertaken as a response to the warning signs involved: surgical pause, irrigation with warm saline, increasing blood pressure and stopping the resection. It is important to note that stopping the resection was not always the goal, as we gave more importance to the neurooncological outcome of patients. This was always communicated with the patients preoperatively in the consent process.

The visual outcome was categorised as stable, deteriorated or improved according to the preoperative and postoperative Goldman campimetry assessments performed by the ophthalmology service [21]. Tumour histology was established according to the CNS WHO classification reviewed in 2021 by a neuropathologist [22].

Statistical analysis was performed with STATA 13.0©. Regression methods (logistic regression—impact of preoperative visual deficit and tractography data of the optic radiations in VF outcomes; multilinear regression—amplitude and latencies from intraoperative neuromonitoring recordings and with visual outcomes; and multinomial regression—impact of tumour location and histology in visual outcomes) were used. A *p*-value < 0.05 was considered significant. We performed ROC analysis to determine what percentage drop in amplitude of the VEPs was significant enough to determine a good correlation with clinical visual outcome.

**Figure 1:**
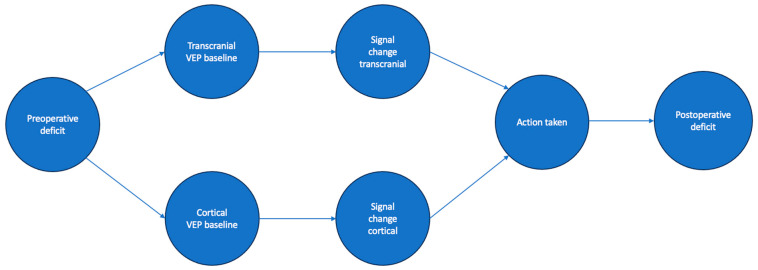
Representation of the Bayesian Network´s nodes and edges.

In addition to the described frequentist statistic methods, we designed a Bayesian network to answer with Bayesian statistics the following questions about our data: In patients who presented with a signal change in surgery, permanent or reversible, how likely was action taken to revert the signal change? What were the postoperative outcome probabilities of these patients? A figure of the network’s nodes and edges can be seen in Figure 1.

## 3. Results

**Figure 2:**
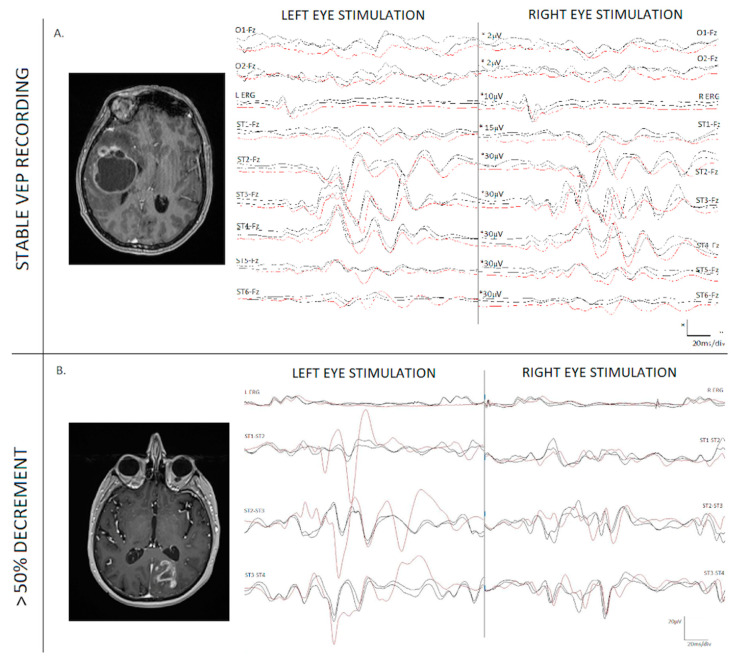
Electroretinogram (ERG) and visual evoked potentials (VEPs) recorded from the strip electrode on two different patients after independent right and left eye stimulation. (**A**) left: Axial T1-weighted MRI image post-gadolinium demonstrating a temporal lesion in the visual eloquent area; (**A**) right: There are no significant changes when comparing the closing recording (black line) with the baseline traces (red line). The patient remained clinically stable; (**B**) left: Axial T1-weighted MRI image post-gadolinium of an occipital lesion invading the visual cortex; (**B**) right: There is a 50% drop in the VEP recordings for left eye stimulation during surgery. This was a warning. The recording improved at the end of the surgery, and the patient’s visual function did not deteriorate.

Thirty-nine patients were included in this study (20 females, 19 males; mean age 52.51 ± 14.08 years old). The most common diagnosis was diffuse intrinsic glioma—24 patients (61.54%), followed by metastasis—11 patients (28.21%). Out of the gliomas, the majority of patients were diagnosed with a WHO grade 4 glioblastoma (70.8%). With regards to their location, there was an almost even distribution between temporal (13 patients), occipital (12 patients) and parietal (10 patients) lobes and a slight predominance of right hemispheric lesions—21 patients (55.26%). Gross total resection (GTR) and supratotal resection (SpTR) were performed in 63.16% of patients (twenty-two and two patients, respectively). Preoperatively, twenty-two patients had no visual deficits; seven patients had either upper or lower quadrantanopia; and ten patients had contralateral homonymous hemianopia. Postoperatively, twenty-nine patients (74.4%) remained stable, nine patients (23.1%) deteriorated, and one patient (2.6%) improved in terms of visual acuity (Figure 2). The presence of a preoperative visual deficit (*p* = 0.3431), the tumour location (*p* = 0.151) and histology (*p* = 0.9240) were not related to the visual outcome after surgery (Table 1).

### 3.1. Preoperative Mapping

**Figure 3:**
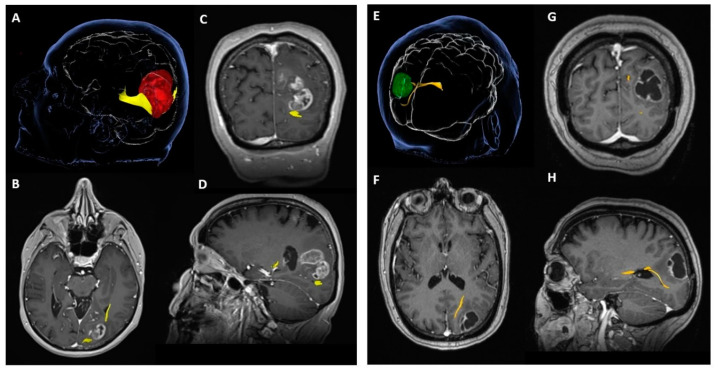
Example of two different tumours in the visual eloquent areas of the brain. (**A**) Probabilistic tractography of the optic radiation, infiltrated by the tumour (tumour—red, optic radiation—yellow); (**B**) axial T1-weightened MRI image post-gadolinium; (**C**) coronal view; (**D**) sagittal view; (**E**) probabilistic tractography of the optic radiation, displaced by the tumour without infiltration (tumour—green, optic radiation—yellow); (**F**) axial T1-weighted MRI image post-gadolinium; (**G**) coronal view; (**H**) sagittal view.

Tractography demonstrated that optic radiation was available for surgical planning in nineteen patients (48.72%) and tract invasion was identified in seven out of nineteen patients (36.8%). The availability of tractography for surgical planning was not related to a better VF outcome after surgery (*p* = 0.287) or a decreased likelihood of VF deterioration after surgery (*p* = 0.449). However, the preoperative documentation of optic radiation infiltration by the tumour (tumour-to-tract distance = 0 mm) was significantly related to VF deficit after surgery (*p* = 0.016) and a higher likelihood of postoperative VF deterioration (*p* = 0.040). (Table 2, Figure 3).

### 3.2. Intraoperative Monitoring

**Figure 4:**
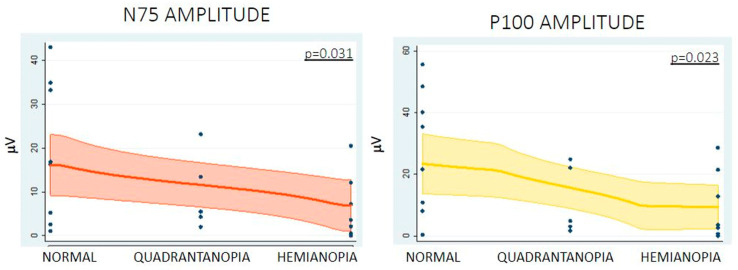
Correlation between N75 and P100 amplitudes at the end of the resection via direct cortical recordings and postoperative visual field deficit.

The relationship between the baseline and closure amplitude and the baseline and closure latency of both N75 and P100 waves of the VEPs using cork screws (transcranial) or strip electrode (direct cortical) recordings for VF outcomes was assessed. No significant statistical correlation was identified between the baseline measures and the preoperative visual deficit or between the measures at closure and the postoperative visual deficits when transcranial recordings were used. A trend towards significance was observed with a higher amplitude of N75 in patients with no preoperative VF deficit—*p* = 0.065 (Supplementary Material Table S1). With regards to direct cortical recording, higher N75 (normal vision 16.77 ± 15.18 µV, quadrantanopia 9.68 ± 8.69 µV, hemianopia 5.29 ± 6.95 µV, *p* = 0.036) and P100 (normal vision 25.11 ± 10.05 µV, quadrantanopia 11.36 ± 11.22 µV, hemianopia 8.71 ± 10.33 µV, *p* = 0.023) amplitudes at closure were associated with the lack of VF deficit after surgery. A similar trend was observed with the N75 baseline amplitude and absence of preoperative VF deficit, even though no statistical significance was reached (*p* = 0.081). (Table 3, Figure 4).

Based on the above-mentioned results regarding the amplitudes of N75 and P100 waves obtained via direct cortical recordings, we assessed the minimal percentage decrease of amplitude related to the VF outcome. Despite using a 50% amplitude drop intraoperatively as a warning, based on published literature [4], we performed a postoperative retrospective ROC analysis. A threshold of 40% deterioration of N75 (*p* = 0.035) and P100 (*p* = 0.020) amplitude was statistically significantly related to a risk of VF deterioration after surgery. For N75, a decrease of 40% in the amplitude has a sensitivity of 80%, a specificity of 77.78%, a positive predictive value (PPV) of 50%, and a negative predictive value (NPV) of 93.33%. For P100, a 40% decrease in the amplitude has the same sensitivity, specificity of 83.33%, PPV of 57.14% and NPV of 93.75%.

### 3.3. Bayesian Analysis

We performed Bayesian analysis on our data, both for the cortical and the transcranial VEP recordings (Supplementary Material Table S2). Regarding the cortical VEPs, given our prior probabilities, when patients did not have preoperative deficits, the following probabilities applied when permanent signal changes were seen: If patients woke up intact, the probability of acting in surgery to revert the signal change was 0.41, whereas if the patients woke up with quadrantanopia, it was 0.22; however, if patients woke up with hemianopia, it was 0.31. The probability that no action was taken was as follows: 0.03, 0.01 and 0.02, respectively, for the postoperative outcomes. Therefore, action would be attempted in the majority of cases, especially in cases when patients woke up intact. In patients with preoperative quadrantanopia that worsened and woke up with hemianopia, action upon the signal change was still more likely (0.31) than no action (0.02).

If we look at the reversible signal changes, in patients with no preop deficit who woke up intact, the probability of acting to revert the signal change was 0.55; in those that woke up with quadrantanopia, it was 0.2; and in those that woke up with hemianopia, it was 0.2. The probabilities of not acting were as follows: 0.03, 0.01 and 0.01, respectively. If a patient presented with quadrantanopia, in cases where the postoperative outcome was worse, developing hemianopia, the likelihood of acting to revert the signal change was 0.32 vs. 0.01 if not acting.

If no signal change was seen with cortical modalities, all patients who presented with normal vision preoperatively woke up intact.

Regarding the transcranial VEP recordings, when patients did not have preoperative deficits, the following probabilities applied when permanent signal changes were seen: If patients woke up intact, the probability of acting in surgery to revert the signal change was 0.37. If the patients woke up with quadrantanopia, it was 0.28. If patients woke up with hemianopia, it was 0.2. The probability that no action was taken was as follows: 0.06, 0.05 and 0.04, respectively, for the postoperative outcomes. Therefore, action would be attempted in the majority of cases, especially in cases when patients woke up intact. In patients with preoperative quadrantanopia that worsened and woke up with hemianopia, action upon the signal change was still more likely (0.28) than no action (0.05).

If we look at the reversible signal changes, in patients with no preop deficit who woke up intact, the probability of acting to revert the signal change was 0.51; in those that woke up with quadrantanopia, it was 0.18; in those that woke up with hemianopia, it was 0.18. The probabilities of not acting were as follows: 0.08, 0.03 and 0.03, respectively. If a patient presented with quadrantanopia, in cases where the postoperative outcome was worse, developing hemianopia, the likelihood of acting to revert the signal change was 0.29 vs. 0.03 if not acting.

If no signal change was seen despite the patient having good baseline signals and no preoperative deficit, the likelihood that the patient would have woken up intact was 0.8. But the likelihood of developing quadrantanopia that was missed by the IOM team was 0.1, and the likelihood of developing hemianopia was 0.1.

## 4. Discussion

This study explores the utility of VEPs in the context of asleep surgery in patients with visual-eloquent tumours. According to our findings, the invasion of the optic radiation, as shown on the preoperative tractography, predicts a poorer visual outcome postoperatively despite the nature of the tumour. From a VEP perspective, higher N75 and P100 amplitudes measured with the subdural strip at closure were associated with better visual outcome postoperatively, and a threshold of 40% deterioration of N75 and P100 amplitudes was related to VF deterioration after surgery. Finally, transcranial VEP monitoring did not correlate significantly with VF outcome.

The nature of the tumour seems not to have had an impact on the outcome. This might indicate that the displacement of the optic radiation (e.g., via a metastatic deposit) might be equally important to the invasion of the tract (e.g., via a glioma). However, our result might not be related to the nature of the tumour, but to surgical manipulation of the tract. In more detail, 11/39 patients in our cohort were operated on due to a metastatic deposit in the parietal or occipital lobe. In these cases, DTI data were used to identify the best approach to the lesion by keeping the OR as intact as possible. In 10/11 patients, the VF outcome remained stable, and the last one even improved postoperatively. This is in keeping with the work of Tanaka et al. in 2021, who demonstrated that complete resection of metastasis via a safe pathway is related to VF improvement [23]. This needs to be further assessed.

VEP monitoring in an asleep setting is reliable, at least in detecting new quadrantanopia [24]. We used two techniques for measuring VEPs in an asleep craniotomy: transcranial recordings using cork screw electrodes and direct cortical recording via strip electrodes. In both methods, an ERG channel is mandatory in order to confirm retinal stimulation. This is supported by the literature that demonstrates that combining ERG with VEP monitoring increases safety and minimises errors during surgery [25].

Direct cortical VEP recordings are not new in the literature. Back in 2010, Ota et al. showed that direct cortical VEP recordings are feasible in epilepsy surgery [20]. Although there were initial waveform variations among their patients, these waveforms remained stable throughout the procedures and exhibited changes that aligned with postoperative visual function, highlighting the potential utility of intraoperative cortical VEP monitoring for assessing the functional integrity of the posterior visual pathway. In 2021, Boëx et al. correlated the latency of the waveforms to the velocity of signal propagation along the optic pathway [9]. They could not demonstrate, however, a rule of thumb to correlate the stimulation intensity to the distance to the optic radiation (similarly to the motor mapping) [26,27]. Carrai et al. demonstrated better stability of the direct cortical recordings but did not record any intraoperative amplitude drop or any postoperative VF worsening [10].

In our study, transcranial recordings were used in the majority of the patients, especially the ones with parietal and temporal tumours, where the positioning of a strip electrode over or close to a calcarine fissure was impossible. Direct cortical recordings were used mainly in occipital tumours that allowed access to the calcarine fissure. However, we used direct cortical recordings even in tumours that were not located within the primary visual cortex or the distal component of the optic radiations (seven patients with temporal lesions and seven with parietal lesions, where direct cortical recording was used). Also, direct cortical recordings were opted for in cases that only allowed suboptimal positioning of transcranial corkscrews.

The invasion of the optic radiation by the tumour can predict the visual outcome of the surgery. Further studies should examine the exact relationship between the distance to the tract and the outcome, as well as the integrity of the tract microstructure as assessed by diffusion imaging data. The location of the tumour, on the other hand, did not correlate significantly with postoperative visual outcome.

Direct cortical recordings in our study had a better correlation with the VF outcome in comparison to the transcranial recordings. An initial suboptimal position of O1/2 electrodes in order to avoid the craniotomy area or even posterior displacement due to skin manipulation/scalp retraction intraoperatively might play a role. To the best of our knowledge, this is the first study demonstrating this finding.

Higher N75 and P100 amplitudes were associated with better visual outcomes postoperatively in our cohort. A wide range in amplitude was noted, which can be explained by the location of the strip in comparison to the calcarine fissure. Nevertheless, amplitude changes have been previously described as a more reliable indicator of neural damage than latency [18]. This could happen for many reasons. For example, latency is more susceptible to changes due to anaesthesia. According to the literature, a significant change in amplitude is defined as a 50% reduction in the N75 and P100 peak-to-peak amplitudes compared to the baseline [11]. In our study, a threshold of 40% deterioration of N75 and P100 amplitude was significantly related to a risk of VF deterioration after surgery. Further studies in larger patient cohorts are needed in order to further clarify the cut-off threshold that should be utilised as an intraoperative warning.

Utilising both continuous VEP monitoring and tractography of the optic radiation proves to be a reliable method for monitoring visual function and assisting in the planning of neurosurgical procedures near the visual pathway [28]. In our series, tractography was performed in 19/39 patients. Even though the existence of tractography did not change the visual outcome in a statistically significant way, it helped us predict VF deterioration when the tumour was invading the optic radiation. This was apparent through the deterioration of the VEPs intraoperatively in these cases. This allows us to assume that by combining the tractography with the intraoperative data of N75 and P100, we can make a better prediction of long-term VF outcomes.

VEP recordings intraoperatively are prone to false warnings. As already demonstrated in existing literature, noise (especially due to muscle artefacts) compromised the quality of recordings. Muscle relaxants can be occasionally used during VEP monitoring, as they do not affect the recordings [29]. In fact, in tumours located in the occipital lobe, where monitoring of the motor-evoked potentials (MEPs) was not essential, our recordings were more reliable due to the use of muscle relaxants. In these cases, we adjusted the use of Rocuronium to a partial muscle blockade with TOF (train of four) at 50%. This concept should be further evaluated with designated studies.

Strip movement during surgery was another factor that affected the recordings, especially in the cases where a tubular retractor was used close to the optic pathway; as part of a minimally invasive parafascicular approach, the recordings were prone to false warnings. Lastly, the level of sedation was related to the quality of the recordings. Total intravenous anaesthesia is essential for reliable VEP monitoring [30].

The results of our Bayesian analysis show interesting outcomes. In most cases, we acted upon signal changes that were seen during the monitoring. The actions taken more frequently were a surgical pause, warm irrigation and increasing blood pressure. Because we valued the oncological outcome of the patients more than the functional outcome, in most cases, the resection was not stopped. Despite this, patients had better outcomes when action was taken to revert the signal changes. This was true even in cases where, despite taking action, the signal change was permanent, especially when using cortical modalities for monitoring. As for the differences in both modalities, it is important to note that in 20% of the cases monitored with transcranial modalities, postoperative deficits would have been seen without a signal change intraoperatively. This raises an issue regarding the sensitivity of transcranial modalities, and we suggest that cortical modalities are used when possible.

### Limitations

The current feasibility study requires validation by external cohorts to enhance the robustness of its findings. Nonetheless, this study enables initial conclusions to be drawn regarding the asleep mapping of the optic radiation and the impact of single-wave analysis (N75 and/or P100). The visual function can be assessed in an awake craniotomy setting given the complexity of the visual assessment, which goes beyond VF determination. However, the included patients were either considered not safe for awake craniotomy from an anaesthetic perspective or decided not to proceed with awake surgery during informed consent when the pros and cons were explained. According to the team’s opinion, this is not unreasonable, as complex visual mapping is not frequently reported as an indication of awake craniotomy in the literature.

It is important to note that our intention was not to compare the effectiveness of intraoperative neuromonitoring in preserving VF but to understand the correlation between intraoperative VEP changes and visual outcomes to improve functional prognostication. Optic radiation tractography was utilised for surgical planning purposes in some cases, but further studies employing advanced tractography algorithms and intraoperative strategies to account for brain shift are needed to enhance the correlation between intraoperative neuromonitoring and tractography. Therefore, we might be able to establish combined risk stratification scores to preserve VF function.

## 5. Conclusions

This study presents findings on the feasibility and reliability of direct cortical monitoring of the primary visual cortex in asleep patients with visual eloquent intra-axial tumours. Direct cortical VEP recordings via strip electrodes demonstrated a strong correlation with visual outcomes and can be a valid alternative to the conventional technique of transcranial recordings in challenging situations. The invasion of the optic radiation is related to worse visual field outcomes. According to Bayesian analysis, taking action upon warning signals was related to a better postoperative outcome, even if the signal changes would not revert intraoperatively.

## Figures and Tables

**Table 1:** Demographic and clinical characteristics of this study’s population.

Demographic and Clinical Characteristics (*n* = 39)	
Sex (Female)	20 (51.28%)
Age (years)	52.51 ± 14.08
Location	
Temporal Occipital Parietal Other	13 12 10 4
Laterality (Right)	21 (55.26%)
Histology	
Glioma Metastasis Other	24 (61.54%) 11 (28.21%) 4 (11.26%)
Diffusion-Weighted Imaging Optic Radiation Invasion	20 (51.28%) 7/20 (35%)
Extent of Resection	
SpTR ^1^ GTR ^2^ STR ^3^	2 (5.26%) 22 (57.89%) 14 (36.84%)
Preoperative Visual Field	
Normal Quadrantanopia Hemianopia	22 (56.41%) 7 (17.95%) 10 (25.64%)
Postoperative Visual Field	
Normal Quadrantanopia Hemianopia	17 (43.59%) 7 (17.95%) 15 (38.46%)
Visual Field Deterioration after Surgery	9 (23.08%)

^1^ GTR: gross total resection. ^2^ SpTR: supratotal resection. ^3^ STR: subtotal resection.

**Table 2:** Univariate analysis of the tractography impact on the optic radiation in the visual field outcome.

	Coefficient	95%CI	*p*-Value
Availability of OR ^1^ for Surgical Planning & Postoperative VF ^2^ Outcome	−0.38 ± 0.36	[−1.09–0.32]	0.287
Availability of OR ^1^ for Surgical Planning & VF ^2^ Deterioration	0.36 ± 0.76	[−1.14–1.85]	0.641
Infiltration of OR ^1^ & Postoperative VF ^2^ Outcome	1.62 ± 0.67	[0.30–2.94]	0.016
Infiltration of OR ^1^ & VF ^2^ Deterioration	2.02 ± 0.99	[0.09–3.96]	0.041

^1^ OR: optic radiation; ^2^ VF: visual field.

**Table 3:** N75 and P100 neurophysiologic properties in direct cortical recordings and visual field outcomes.

N75 and P100 Neurophysiologic Properties and Visual Field Outcomes			
	Coefficient	95%CI	*p*-Value
**Baseline**			
**N75 Latency**	−3.92 ± 0.5.67	[−15.68–7.84]	0.497
**N75 Amplitude**	−5.65 ± 3.08	[−12.04–0.75]	0.081
**P100 Latency**	−5.18 ± 6.63	[−18.92–8.56]	0.443
**P100 Amplitude**	−6.49 ± 4.00	[−14.80–1.82]	0.120
**Closure**			
**N75 Latency**	−7.04 ± 5.09	[−17.60–3.51]	0.180
**N75 Amplitude**	−5.75 ± 2.58	[−11.10–−0.41]	0.036
**P100 Latency**	−8.33 ± 7.85	[−24.61–7.95]	0.300
**P100 Amplitude**	−8.26 ± 3.37	[−15.26–−1.26]	0.023

## Data Availability

All relevant data are included in the paper. The rest of the data are not available due to privacy reasons.

## Supplementary Materials

The following supporting information can be downloaded at: https://www.mdpi.com/article/10.3390/cancers15153943/s1, Table S1: N75 and P100 amplitudes and latencies and Visual Field Outcomes with transcranial VEP recording; Table S2: Results of the Bayesian analysis performed in patients with no preoperative deficits.
