# Supplementary material for: Intraoperative Neuromonitoring of the Visual Pathway in Asleep Neuro-Oncology Surgery
Source: Cancers (Basel). 2023 Aug 3;15(15):3943. doi: 10.3390/cancers15153943 (PMC10416823; doi:10.3390/cancers15153943)
Supplement: Supplementary file 1 [file cancers-15-03943-s001.zip › cancers-2460116-SI.pdf]

Table S1. N75 and P100 amplitudes and latencies and Visual Field Outcomes with transcranial VEP recording.

Transcranial (Cork Screw) VEP Recording			
Baseline			
	Coefficient	95%CI	*p*-value
N75 Latency	−4.13 ± 2.87	[−9.99–1.74]	0.161
N75 Amplitude	0.74 ± 0.39	[−0.05–1.53]	0.065
P100 Latency	2.09 ± 4.58	[−7.25–11.43]	0.652
P100 Amplitude	7.06 ± 4.50	[−2.11–16.24]	0.127
Closure			
	Coefficient	95%CI	*p*-value
N75 Latency	−5.52 ± 4.27	[−14.21–3.17]	0.205
N75 Amplitude	0.41 ± 0.37	[−0.34–1.16]	0.270
P100 Latency	−8.22 ± 7.03	[−22.54–6.10]	0.251
P100 Amplitude	0.22 ± 0.31	[−0.40–0.85]	0.470

Table S2. Results of the Bayesian analysis performed in patients with no preoperative deficits. It shows the probabilities of waking up intact/with a new-onset visual deficit for patients when action was taken/not taken to revert the signal changes seen on these IONMs. We compare direct cortical VEPs with transcranial VEPs and permanent signal changes with reversible signal changes.

Bayesian Analysis for Direct Cortical and Transcranial VEP Recordings				
Direct Cortical VEPs	Permanent Signal Change		Reversible Signal Change	
	Action	No Action	Action	No Action
No Deficit	0.41	0.03	0.55	0.03
Quadrantanopia	0.22	0.01	0.2	0.01
Hemianopia	0.31	0.02	0.2	0.01
Transcranial VEPs	Permanent Signal Change		Reversible Signal Change	
	Action	No Action	Action	No Action
No Deficit	0.37	0.06	0.51	0.08
Quadrantanopia	0.28	0.05	0.18	0.03
Hemianopia	0.2	0.04	0.18	0.03
